# Simultaneous Corneal Topography and Epithelial Thickness Mapping from a Single Measurement Using Optical Coherence Tomography

**DOI:** 10.1155/2022/7339306

**Published:** 2022-04-21

**Authors:** Bartosz L. Sikorski

**Affiliations:** ^1^Department of Ophthalmology, Nicolaus Copernicus University, 9 M. Sklodowskiej-Curie Street, Bydgoszcz 85-309, Poland; ^2^Oculomedica Eye Research and Development Centre, Ogrody 14 Street, Bydgoszcz 85-870, Poland

## Abstract

**Purpose:**

To evaluate the performance of corneal epithelial thickness mapping (ETM) and demonstrate simultaneous measurement of ETMs and corneal topography using REVO NX (Optopol Technology, Zawiercie, Poland)—an OCT device for anterior and posterior segment imaging.

**Methods:**

One hundred thirty-seven eyes of 137 normal subjects and patients with corneal diseases were recruited to the study. Each subject was scanned with REVO NX. ETMs and corneal topography maps were reconstructed from a single measurement. Corneal topography was also carried out using Pentacam (Oculus, Wetzlar, Germany). One hundred twenty-eight eyes were qualified for the final analysis. Forty healthy eyes were used to evaluate the performance of ETM, and 88 eyes were used to compare ETMs and corneal topography. The repeatability and reproducibility of ETMs in healthy subjects were assessed on the basis of 17 spatial zones derived from an 8-mm diameter corneal scan using within-subject standard deviation, test-retest repeatability, within-subject coefficient of variation (CoV), and intraclass correlation coefficient (ICC).

**Results:**

The ICC for both repeatability and reproducibility of ETMs for the central sector was 0.95. The ICC value for the other sectors was only moderately lower. However, the CoV for repeatability (≤1.55%) was slightly higher than the value reported for the RTVue device (Optovue, Inc, Fremont, California, USA), for which a CoV in the central zone of 1.07% was reported in unoperated eyes. The superior quadrants were found to be the thinnest while the inferior ones were the thickest. ETMs and topography maps created from a single OCT measurement present a complementary image of the cornea.

**Conclusions:**

ETMs obtained using REVO NX show high levels of repeatability and reproducibility in normal eyes. Because the topographic and epithelial thickness analyses are performed using the same data, which means they are based on the exact same 3D corneal model, they do not require reciprocal centration and map matching. This ensures a complete point-to-point correlation between ETMs and corneal topography maps, which paints a fuller picture of a given pathology.

## 1. Introduction

Accurate corneal imaging is critical for both diagnosis and effective management and treatment of patients with ocular surface diseases. As new diagnostic imaging modalities become more detailed and treatments for many eye diseases become more sophisticated, the ability to obtain a more complete picture of a condition through multimodal imaging becomes particularly important. In recent years, with the popularization of spectral domain optical coherence tomography (OCT), we have gained the ability to routinely perform corneal epithelial thickness mapping (ETM) in daily clinical practice, in addition to the classic OCT visualization of the corneal structure and corneal curvature and power in topography. The first commercially available device capable of creating ETMs was the RTVue 100 (Optovue Inc., Fremont, California, USA). The device demonstrated excellent repeatability of corneal epithelial thickness measurements in normal as well as in keratoconus (KCN) eyes, dry eye patients, and postlaser-assisted in situ keratomileusis eyes [1–4]. ETMs were also shown to be helpful in differentiation KCN from contact lens warpage in patients with abnormal topography [5]. The RTVue, however, did not have the capacity to perform corneal topography, which could be correlated with an ETM to obtain a more complete picture of the pathology. The latest version of the device, Avanti (Optovue Inc., Fremont, California, USA), does not offer this possibility either. In order to compare ETMs with corneal topography, one needs to use data from other diagnostic devices, which brings the risk of nonideal spatial and temporal correlation of examinations. Recently, a novel stand-alone anterior segment OCT device, which provides the ability to perform imaging of the anterior segment and obtain ETMs combined with placido disc corneal topography, has been introduced into clinical practice (MS-39, CSO, Firenze, Italy) [6]. It delivers high repeatability of ETM measurements in healthy and KCN eyes [7]. However, as mentioned earlier, the device does not use the OCT technique to create corneal topography maps and thus also uses different data than those used to plot ETMs. Anterior segment-only OCTs using swept-source (SS-OCT) technology, such as the Casia 2 (Tomey Corporation, Nagoya, Japan) and Anterion (Heidelberg Engineering GmbH, Heidelberg, Germany) devices, are also commercially available for corneal topography. However, they have not released their ETM capabilities that are currently under clinical trials. This function is present only in its investigational software version and has not been released commercially [8].

The first device to offer full commercial corneal topography and ETMs derived from an OCT examination is REVO NX (Optopol Technology, Zawiercie, Poland). The device also gives the possibility to create topographic maps and ETMs from one data set obtained in a single measurement. The goal of this study is to evaluate the performance of ETMs and to demonstrate the implementation of ETM measurement with a simultaneous registration of corneal topography in the REVO NX device.

## 2. Methods

### 2.1. Subjects

The study was conducted from May 2020 to June 2021 and included 137 subjects (mean age: 41.4 ± 14.5 years; 65 females). Subjects showing no changes in the corneal structure in an examination using a slit lamp as well as patients with corneal diseases were qualified for the study. A complete ocular assessment was performed. All patients were examined using REVO NX (Optopol Technology S.A, Zawiercie, Poland) with the topography OCT (T-OCT) module with an add-on anterior adapter (software version 9.5). Topography maps and ETMs were produced using the topography protocol, which is a modified 8-mm anterior radial scanning protocol. During the examination, the center of the pupil was positioned at the center of the measurement window to keep the central reflection centered in both the horizontal and vertical preview windows of the tomogram. Participants were asked to blink to enable corneal tear film coverage before measurements and to concentrate on the fixation target during the scan and open the eye widely. Each measurement was checked for quality. If it did not meet the manufacturer's recommended total quality factor (TQF) value, the test was rejected and a new measurement was performed. The procedure was repeated up to three times. If the quality of all measurements was not good enough, the eye was excluded from the analysis. Topography maps were also made for all patients using Pentacam AXL (Oculus, Wetzlar, Germany). Like in the case of REVO NX, the measurement was also repeated three times, if it did not meet the quality criteria. Maps of good quality were obtained on both devices in 88 of 97 subjects (the most common reason for examination rejection was lack of stable fixation or dropping eyelid). This included 32 healthy eyes (mean anterior K1, K2, and cylinder for REVO NX were 42.71 ± 1.05 D, 43.95 ± 1.30 D, and 1.20 ± 0.99 D, respectively; mean anterior K1, K2, and cylinder for Pentacam were 42.77 ± 0.99 D, 44.07 ± 1.35 D, and 1.31 ± 1.05 D, respectively; mean posterior K1, K2, and cylinder for REVO NX were −6.02 ± 0.27, −6.37 ± 0.33, and 0.35 ± 0.19, respectively; and mean posterior K1, K2, and cylinder for Pentacam were −6.10 ± 0.20, −6.47 ± 0.30, 0.39 ± 0.18, respectively) and 56 eyes with corneal pathologies (mean anterior K1, K2, and cylinder for REVO NX were 44.36 ± 5.61 D, 47.34 ± 7.51 D, and 3.00 ± 2.96 D, respectively; mean anterior K1, K2, and cylinder for Pentacam were 44.25 ± 5.58 D, 47.55 ± 7.79 D, and 3.29 ± 3.52 D, respectively; mean posterior K1, K2, and cylinder for REVO NX were −5.99 ± 0.92, −6.90 ± 1.35, and 0.93 ± 0.69, respectively; and mean posterior K1, K2, and cylinder for Pentacam were −6.19 ± 1.27, −6.81 ± 1.61, and 0.63 ± 0.63, respectively). Additionally, 40 healthy eyes (40 healthy volunteers, mean age 35.7 ± 8.38 years) were examined only with REVO NX in order to determine intraobserver repeatability and interobserver reproducibility of ETMs. Eight of these subjects had astigmatism <0.5 D, 16 subjects had astigmatism in the range of 0.5–1.0 D, and 16 subjects had astigmatism exceeding 1 D. Mean K1 and K2 [D] were 43.26 ± 0.99 and 44.46 ± 1.73, respectively. The values of K1 and K2 [mm] were 7.8 ± 0.18 and 7.6 ± 0.28, respectively. The cylinder [D] value was 1.2 ± 1.24 (with a range of: 0.2–7.6). The respective values of Min K [D] and Avg K [D] were 44.58 ± 1.76 and 43.85 ± 1.25 (with a range of 42.56–47.80). The respective values of Min K [D] and Avg K [D] were 7.58 ± 0.27 and 7.70 ± 0.20 (with a range of 7.06–7.93). Each measurement was taken three times by 2 operators in a random order, under ambient lighting conditions. Following each measurement, the subject head was repositioned on the chin rest and the REVO NX device was realigned. The study protocol was in accordance with the Declaration of Helsinki. The Institutional Ethics Committee approval was granted, and informed consent was obtained from all participants.

### 2.2. Creation of Topography Maps and ETMs

Topography maps and ETMs were reconstructed using REVO NX with the anterior adapter. It is an OCT device dedicated to posterior as well as anterior segment imaging, with the ability to analyze corneal curvature and a built-in optical biometry module using the split-window OCT method [9]. The device has the following technical parameters: the LED light source with central wavelengths of 830 nm, a scanning speed of 110 000 A-scan/sec, a scan depth window of 2.43 mm, a maximum anterior scan width of 16 mm, an axial resolution of 5 *μ*m (digital∼2,5 *μ*m), and the transverse resolution of 18 *μ*m.

**Figure 1:**
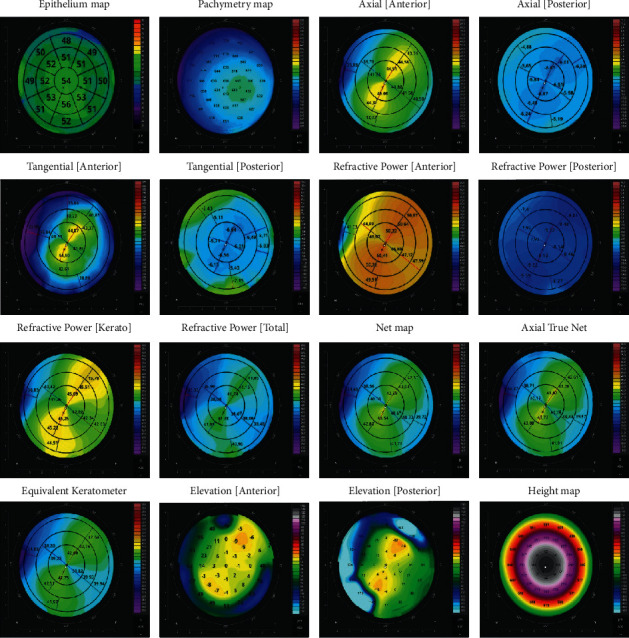
Topography maps and ETMs generated during a simultaneous measurement by REVO NX with 16 evenly spaced meridian B-scans of 8-mm width. To the right of each map are the color scales used to represent the analyzed parameter.

Topography maps and ETMs were produced using the topography protocol, which is a modified anterior radial scanning protocol. It consists of 16 evenly spaced meridian B-scans (8 mm in length each). Every B-scan consists of 1024 A-scans. The examination time is 0.17 sec. After collecting the data, the algorithm automatically recognizes the B-scans' 3 boundaries defining the edges of corneal layers: air and corneal boundary, the posterior epithelium boundary, and the posterior endothelium boundary, which makes it possible to create a thickness map of the whole cornea, epithelium, and stroma. The posterior border of the epithelium is detected as the border of the epithelium and the Bowman membrane. In the absence of the Bowman membrane, for example, in patients who have undergone photorefractive keratectomy, the posterior border is defined as the border of the corneal epithelium and stroma. Once the layers are recognized, the system dewarps the collected tomograms to reconstruct the actual shape and profile of the anterior and posterior corneal surfaces. Next, a 3D model of the corneal surface is created on the basis of the B-scans and the identified layers. The reconstructed corneal profile undergoes a qualitative assessment expressed as a summary TQF that determines whether the operator can trust the measurement. The TQF is based on the values of the following individual factors: the quality index, which is based on the signal-to-noise ratio of all tomograms, and the correlation index, which describes the quality of the tomogram correlation and the analyzed area index that indicates the percentage of the recognized area for anterior and posterior surfaces of the cornea. Metrics describing corneal curvature, topography maps, and ETMs are not displayed for scans with poor quality or with motion artifacts that cannot be compensated. The software also detects imaging artifacts and displays adequate warnings whenever on 3 consecutive scans. The system detects areas where there is no continuous corneal structure and the corneal layers cannot be recognized. This makes it easy to recognize artifacts caused by a closed eyelid, a ghost signal from the iris, a lack of signal due to long eyelashes, or a weak signal due to corneal opacity. If the quality of data is insufficient for analysis, the system does not display the data or marks the area as interpolated with a texture of different transparency. The corneal vertex is determined on the 3D model, which is followed by the creation of 16 maps describing the curvature and thickness of the cornea in relation to the vertex (Figure 1). The maps include the following: Axial map [Anterior], Axial map [Posterior], Tangential map [Anterior], Tangential map [Posterior], Refractive Power map [Anterior], Refractive Power map [Posterior], Refractive Power map [Kerato], Refractive Power map [Total], Net map, Axial True Net, Equivalent Keratometer map, Elevation map [Anterior], Elevation Map [Posterior], Height map, and Pachymetry map. An ETM is also created on the basis of the same data and the 3D model of the cornea. Quantitative data for topography maps are displayed in 3 zones, i.e., 3 mm, 5 mm, and 7 mm. Also, the simulated keratometry value (Sim K) is calculated for the anterior and posterior corneal surfaces, and real power is calculated according to the thick lens formula. ETMs display an averaged epithelial thickness across 17 sectors. Around the central sector with the diameter of 2 mm, the following paracentral sectors are found: Superior, Superior-temporal, Temporal, Inferior-temporal, Inferior, Inferior-nasal, Nasal, and Superior-nasal. Outwards from these, there are the following sectors: Superior_Out, Superior-temporal_Out, Temporal_Out, Inferior-temporal_Out, Inferior_Out, Inferior-nasal_Out, Nasal_Out, or Superior-nasal_Out. The limits of the zones outside the central 2-mm sector are 2–5 mm and 5–7 mm, respectively.

### 2.3. Statistical Analysis

Statistical analysis was performed using Statistica 13.1 software (Dell Inc., USA). To determine the intraobserver repeatability and interobserver reproducibility of the REVO ETMs, the within-subject standard deviation (Sw), test-retest repeatability (TRT), coefficient of variation (CoV), and intraclass correlation coefficient (ICC) were calculated and analyzed. The Sw was the square root of the residual mean square in the one-way analysis of variance. The TRT was defined as 2.77 Sw, which shows the interval within which 95% of the differences between measurements are expected to lie [10]. The percentage of CoV was calculated as the ratio of the Sw to the mean. The ICC represented the consistency in data measurement: high agreement is indicated by a value higher than 0.9 [11]. To assess agreement between REVO NX and Pentacam AXL topography for anterior K1, anterior K2, anterior cylinder, posterior K1, posterior K2, and posterior cylinder, the paired samples *t*-test and Bland–Altman analyses were performed, and 95% limits of agreement were calculated by the mean difference ± 1.96 SD [12]. A *p* value of less than 0.05 was considered statistically significant.

## 3. Results

### 3.1. Repeatability and Reproducibility of ETMs

The results of intraobserver repeatability and interobserver reproducibility assessment in 17 sectors of ETMs using REVO NX in 40 healthy eyes are shown in Tables 1 and 2. The ICC of repeatability for the central sector was ≥0.95. The ICC values for the sectors immediately surrounding the central sector were the following: Superior ≥0.88, Superior-temporal ≥0.82, Temporal ≥0.86, Inferior-temporal ≥0.91, Inferior ≥0.89, Inferior-nasal ≥0.85, Nasal ≥0.91, and Superior-nasal ≥0.88. The ICC value for the peripheral sectors were as follows: Superior_Out ≥0.76, Superior-temporal_Out ≥0.79, Temporal_Out ≥0.69, Inferior-temporal Out ≥0.74, Inferior_Out ≥0.81, Inferior-nasal_Out 0.79, Nasal_Out ≥0.89, and Superior-nasal_Out ≥0.64. The value of the ICC of reproducibility for the central sector was 0.95, while for the inner sectors, it showed the following values: Superior=0.92, Superior-temporal=0.87, Temporal=0.90, Inferior-temporal=0.91, Inferior=0.90, Inferior-nasal=0.87, Nasal=0.91, Superior-nasal=0.86, Superior_Out=0.76, Superior-temporal_Out=0.77, Temporal_Out=0.69, Inferior-temporal_Out=0.77, Inferior_Out=0.77, Inferior-nasal_Out=0.79, Nasal_Out=0.77, and Superior-nasal_Out=0.60.

### 3.2. Clinical Cases

The comparison of the ETMs and topography maps obtained using REVO NX and Pentacam AXL was based on a group of 88 patients. The mean anterior K1 [D] measurement difference in 32 healthy eyes was −0.06 ± 0.15 (*p*=0.09, the 95% limits of agreement (LoA) on Bland–Altman plots ranged from −0.35 to 0.25); the mean anterior K2 [D] difference was −0.12 ± 0.26 (*p*=0.05, 95% LoA −0.60 to 0.40); the mean anterior cylinder [D] difference was −0.12 ± 0.26 D (*p*=0.05, 95% LoA −0.60 to 0.40); the mean posterior K1 [D] difference was 0.08 ± 0.18 D (*p*=0.05, 95% LoA −0.25 to 0.45); the mean posterior K2 [D] difference was 0.10 ± 0.24 (*p*=0.06, 95% LoA −0.35 to 0.60); and the mean posterior cylinder [D] difference was −0.04 ± 0.08 (*p*=0.04, 95% LoA −0.20 to 0.10). The mean anterior K1 [D] measurement difference in 56 eyes with corneal diseases was 0.11 ± 0.22 (*p*=0.05, 95% LoA −0.30 to 0.50); the mean anterior K2 [D] difference was −0.21 ± 0.87 (*p*=0.17, 95% LoA −1.90 to 1.50); the mean anterior cylinder [D] difference was −0.29 ± 0.78 (*p*=0.08, 95% LoA −1.80 to 1.25); the mean posterior K1[D] difference was 0.20 ± 0.70 (*p*=0.44, 95% LoA −1.20 to 1.60); the mean posterior K2 [D] difference was −0.09 ± 0.59 (*p*=0.69, 95% LoA −1.25 to 1.05); and the mean posterior cylinder [D] difference was 0.30 ± 0.52 (*p*=0.15, 95% LoA −0.75 to 1.35).

**Figure 2:**
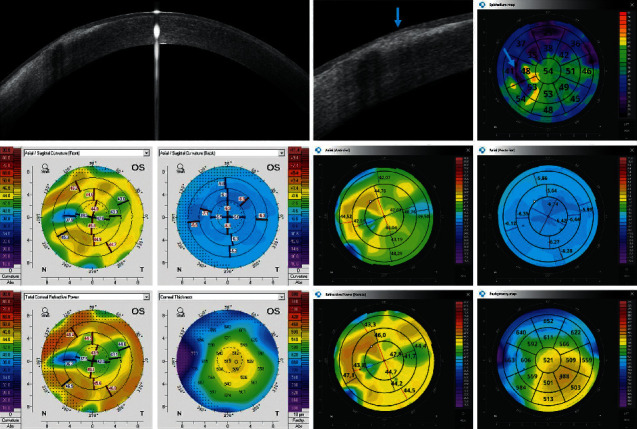
Postpterygium surgery OCT tomogram shows an irregular reduction in the epithelial thickness across the entire upper and partially nasal portions of the central cornea on the ETM (blue arrows). Topography maps reveal irregular astigmatism of the anterior surface of the cornea while the posterior surface is regular. The measurements performed with Pentacam have a white background, and the ones performed with REVO NX have a black background. The K1, K2, and astigmatism values for the anterior surface of the cornea obtained using Pentacam are 41.2 D, 44.8 D, and 3.6 D, respectively, while the values obtained using REVO NX are 41.4 D, 44.5 D, and 3.1 D, respectively. The values of analogous parameters for the posterior corneal surface from Pentacam are −6.5 D, −6.6 D, and 0.1 D and the values from REVO NX are −6.2 D −6.5 D 0.3 D.

**Figure 3:**
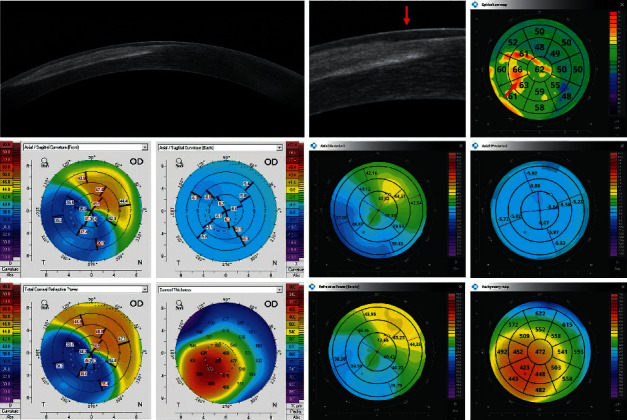
Ulcerative keratitis with extensive corneal stromal scarring. ETM shows thickening of the epithelium in the area of the disease process (red arrow). Topography maps depict irregular astigmatism of the anterior surface of the cornea while the posterior surface is regular. The measurements performed with Pentacam have a white background, and the ones performed with the REVO NX have a black background. The K1, K2, and astigmatism values for the anterior surface of the cornea obtained using Pentacam are 36.8 D, 42.9 D, and 6.1 D, respectively, while the values obtained using REVO NX are 37.6 D, 42.9 D, and 5.3 D, respectively. The values of analogous parameters for the posterior corneal surface from Pentacam are −6.0 D, −6.3 D, and 0.3 D and the values from the REVO NX are −5.8 D, −6.0 D, and 0.2 D.

**Figure 4:**
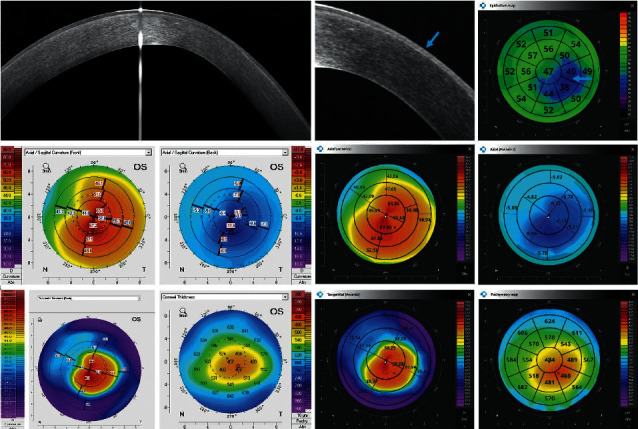
Cornea with keratoconus. The decrease in corneal epithelial thickness (blue arrow) corresponds to the increase in corneal thickness on the pachymetry map. Topography maps show that both the anterior and posterior corneal surfaces have irregular curvature. The measurements performed with Pentacam have a white background, and the ones performed with the REVO NX have a black background. The K1, K2, and astigmatism values for the anterior surface of the cornea obtained using Pentacam are 51.0 D, 62.9 D, and 11.9 D, respectively, while the values obtained using the REVO NX are 50.9 D, 61.9 D, and 11.0 D, respectively. The values of analogous parameters for the posterior corneal surface from Pentacam are −7.7 D, −9.7 D, and 2.0 D and the values from REVO NX are −7.2 D, −9.4 D, and 2.2 D.

**Figure 5:**
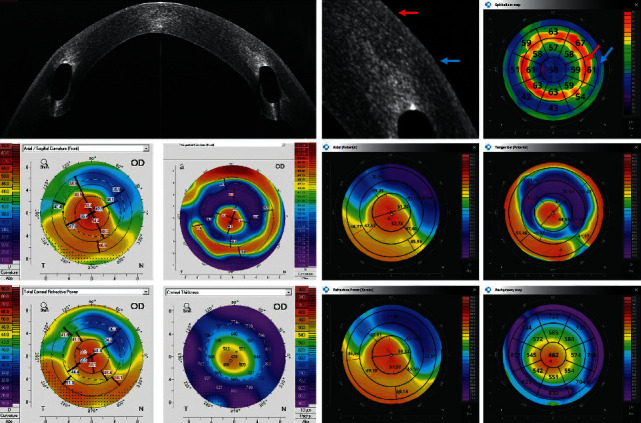
Cornea with an intracorneal ring segment. OCT reveals epithelial thinning immediately above the ring (blue arrow) with the thickness increasing toward the ring (red arrow). Topography maps reveal abnormalities in the anterior surface of the cornea. The measurements performed with Pentacam have a white background, and the ones performed with REVO NX have a black background. The K1, K2, and astigmatism values for the anterior surface of the cornea obtained using Pentacam are 48.9 D, 52.8 D, and 4.0 D, respectively, while the values obtained using REVO NX are 48.6 D, 52.4 D, and 3.8 D, respectively. The values of analogous parameters for the posterior corneal surface from Pentacam are −9.1 D, −9.5 D, and 0.3 D and the values from REVO NX are −9.1 D, −9.7 D, and 0.6 D.

**Figure 6:**
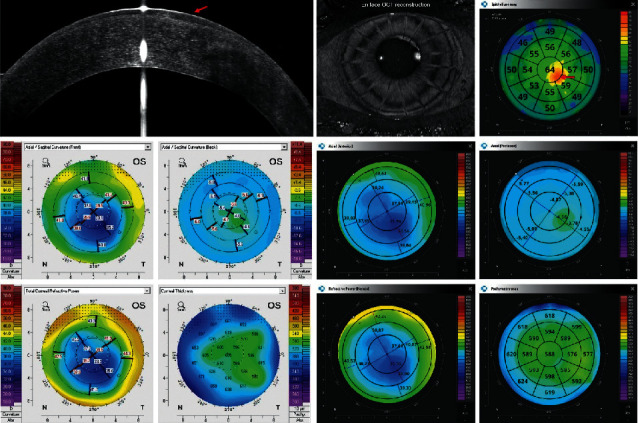
Postradial keratotomy eye. ETM shows a small paracentral island of increased epithelial thickness (red arrow). Topography maps show an area of reduced curvature of the posterior corneal surface at this location. Also, a flattening of the anterior surface of the cornea can be observed. The measurements performed with Pentacam have a white background, and the ones performed with REVO NX have a black background. The K1, K2, and astigmatism values for the anterior surface of the cornea obtained using Pentacam are 35.5 D, 37.5 D, and 1.9 D, respectively, while the values obtained using REVO NX are 35.6 D, 37.5 D, and 1.9 D, respectively. The values of analogous parameters for the posterior corneal surface from Pentacam are −4.5 D, −5.0 D, and 0.5 D and the values from the REVO NX are −4.2 D, −5.0 D, and 0.8 D.

**Figure 7:**
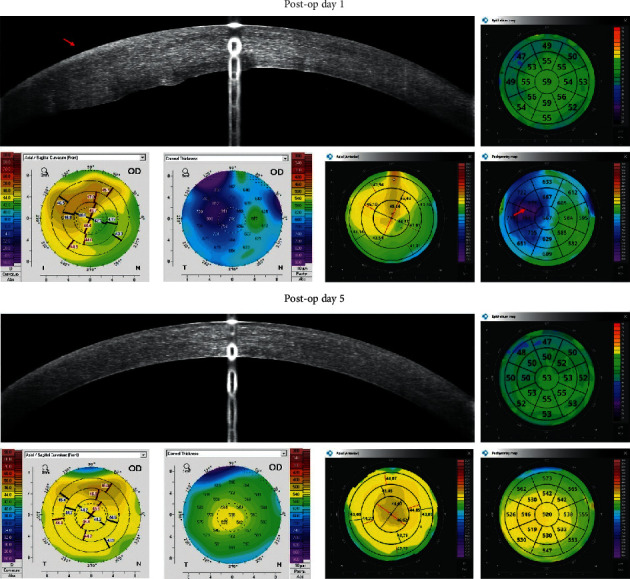
Condition after cataract surgery. On postop day 1, corneal oedema is visible on the pachymetry map (red arrow) and a disruption of the regularity of the anterior corneal surface can be observed on the result of corneal topography. On postop day 5, the oedema regressed. Despite a significant change in the corneal thickness and curvature between examinations, ETMs show a similar image. The measurements performed with Pentacam have a white background, and the ones performed with REVO NX have a black background.

Figures 2–7 show representative examples of corneal pathologies, selected from the group of eyes mentioned above, imaged with REVO NX using simultaneous corneal topography (dark background) and ETMs from a single measurement together with corresponding topography maps created using Pentacam (white background).

Figure 2 shows the condition after pterygium surgery that resulted in an irregular astigmatism. An OCT tomogram depicts an irregular reduction in the epithelial thickness across the entire upper and partially nasal portions of the central cornea on the ETM (blue arrows). The Axial Anterior and Refractive Power maps reveal that the anterior surface of the cornea is damaged and the posterior surface is regular. In the area of the removed pterygium, the cornea is thicker due to scarring, as shown by pachymetry. The same relationship is also evident on maps created using Pentacam.

Figure 3 shows ulcerative keratitis with extensive corneal stromal scarring. In the area of the disease process, ETM reveals a thickening of the epithelium (red arrow) accompanied by an increase in corneal thickness on the pachymetry map. Again, the Axial Anterior and Refractive Power maps demonstrate that the patient's irregular astigmatism originates from the anterior corneal surface. The posterior corneal surface is normal. Corneal topography performed with Pentacam shows similar results.

Figure 4 shows the ocular surfaces of a patient with corneal ectasia. The decrease in corneal epithelial thickness (blue arrow) corresponds to the increase in corneal thickness on the pachymetry map in a manner typical for KNC. Axial Anterior and Tangential Anterior as well as Tangential Posterior maps show that both the anterior and posterior corneal surfaces have irregular curvature. They are represented in the same way on the Pentacam maps.

The example in Figure 5 shows an intracorneal ring segment. OCT reveals epithelial thinning immediately above the ring (blue arrow), with the thickness increasing toward the ring (red arrow). This relationship is clearly visible in the en face image on the ETM. The Axial Anterior, Tangential Anterior, and Refractive Power maps show anterior corneal surface abnormalities, which correlate well with the results from Pentacam.

Figure 6 shows a postradial keratotomy eye. The OCT en face reconstruction depicts corneal incisions spreading radially. The Axial Anterior and Refractive Power maps show the flattening of the central cornea that is typical for this procedure. A similar pattern can be observed on the Pentacam maps. Both devices present a small paracentral island with the reduced curvature of the posterior corneal surface. On the ETM, one can observe an increase in epithelial thickness (red arrow) exactly in the same place (red arrow).

The final example shows the patient's eye on the first and fifth days after cataract surgery (Figure 7). At the first follow-up visit, one can observe a large corneal oedema on the pachymetry map (red arrow) and an irregularity of the anterior corneal surface on the Axial Anterior map. At the next visit, the oedema is no longer visible. Despite a significant change in the corneal thickness and curvature between examinations, ETMs show a similar image.

## 4. Discussion

The analysis of the distribution of the epithelial thickness is a valuable diagnostic and follow-up tool when dealing with patients presenting corneal abnormalities caused by such factors as the presence of pathogenic changes, wearing contact lenses, or undergoing refractive surgeries. For this reason, ETMs have been increasingly used in recent years to evaluate the ocular surface. It can be assumed, therefore, that as new devices featuring ETM functionality come on the market, its popularity will continue to increase. Since the diagnostic value of ETMs is more complete when combined with corneal topography (which provides complementary information about the ocular surface), it would be very practical to have a tool capable of performing both examinations simultaneously. However, the OCT devices available on the market provide either ETMs (Avanti, Optovue Inc., Fremont, California, USA; MS-39, CSO, Firenze, Italy) or topography maps (Heidelberg Engineering GmbH, Heidelberg, Germany; Casia 2, Tomey Corporation, Nagoya, Japan). The world's first commercially available OCT device that can perform a simultaneous measurement of both epithelial thickness and corneal topography is REVO NX, and it is not a trivial task.

Performing reliable ETMs using commercially available OCT devices can be difficult due to several aspects. First of all, the corneal epithelium has a relatively small thickness in relation to the optical axial resolution of OCT devices. The majority of devices on the market that operate at the wavelengths in the range of 830–850 nm typically features an optical axial resolution of around 5 *μ*m (e.g., REVO NX), effectively delivering digital image resolution of around 2.5 *μ*m. What is more, the transverse resolution of OCT devices is much lower than their axial resolution, which makes it more difficult to visualize small and local changes in epithelial thickness. This problem can become even more pronounced in SS-OCT devices, which offer less axial resolution due to their longer wavelength. Also, the low reflectivity of the boundary between the epithelium and the Bowman membrane can make it difficult to properly segment the corneal layers. When this boundary disappears, only the interface between the epithelium and the stroma is visible. Scanning the cornea several times at the same locations and averaging the tomograms may be helpful to overcome the difficulties described above. This, however, increases scanning time and can lead to difficulties in building correlations between consecutive B-scans, between which involuntary eye movements occur [13].

Also, OCT topography methods must overcome a challenge connected with the movement of the eye that can occur during serial image acquisition. Even during fixation, eye movements, such as drift and microsaccades, occur [14]. Especially microsaccades, which are larger (0–2 deg) and more rapid (∼40 deg/s) eye movements occurring at a rate of 1–2 times per second, if not handled properly, can cause significant distortions in topography maps and adversely impact measurement accuracy and repeatability [13, 15, 16]. One should also keep in mind that building a reliable 3D model of the cornea and performing its spatial alignment, which is essential for creating topography maps, is particularly difficult when it is based on data from OCT devices dedicated to posterior segment imaging. Apart from REVO NX, no other such commercially available OCT device offers the functionality of corneal topography. The Avanti device in the corneal power scan mode (8 radial scans with 6-mm scan length of 1024 A-scans) only provides mean Net, Anterior, and Posterior power measurements, representing the net corneal power, anterior corneal power, and posterior corneal power, respectively. The corneal curvature radii, on the other hand, are derived based only on the best fit sphere to the central 3 mm for anterior and posterior surfaces.

Since topography maps and ETMs are generated by REVO NX from exactly the same data set and are based on the same 3D corneal model, the spatial correlation between them is complete, enabling a point-to-point comparison. As shown in the presented clinical examples (Figures 2–7), ETMs obtained in this way are perfectly complementary to topography maps. For example, in Figure 6, we can see a nonobvious correlation of an area of increased epithelial thickness with an island of decreased curvature of the posterior corneal surface. The topography maps obtained with REVO NX correspond well with the topography maps from Pentacam. When ETMs and topography maps are created from different OCT examinations, there is a risk of a nonideal fit and centration of the examinations as well as differences in the two 3D corneal models.

It should be mentioned that REVO NX also features the anterior radial scan functionality for more precise reproduction of the epithelial course on single B-scans. It includes a smaller number of tomograms, which are 7 mm wide (8 B-scans versus 16 B-scans) but their resolution is higher (2560 A-scans), and they are repeated 3 times. In this case, therefore, ETMs and topography maps are created from different data. With more A-scans and several repetitions of the scan at a single location (averaged B-scans), the visibility of the epithelium-Bowman membrane boundary is improved, making it easier to detect the corneal layers. The downside of this scanning protocol is the longer measurement time and lower angular density of the scan, potentially reducing the sensitivity of ETMs in the visualization of small local changes within the epithelium. The protocol is similar to the one of the Avanti device.

The ICC index for ETMs obtained with REVO NX in this study, for both intraobserver repeatability and interobserver reproducibility, has lower values for the outer sectors. This may be connected with the decreasing precision of epithelial thickness measurements as one moves away from the center of the cornea due to a weaker signal at the periphery of the image. It is difficult to directly compare the obtained results with the results from other devices due to the different area of the sectors analyzed. In the case of the MS-39 device, the authors evaluated the repeatability of the central epithelial thickness reading over an area of 3.0 mm and the 4 paracentral measurements (nasal, temporal, superior, and inferior), with a diameter between 3.0 mm and 6.0 mm. However, for the central sector (which is larger than in REVO NX), they obtained an ICC value of 0.957 and CoV on the level of 1.87%. [6] The values are similar to the ones obtained in this study (ICC ≥ 0.95; CoV ≤ 1.55%). The ICC value for the other sectors was only slightly lower. However, the CoV was slightly higher than the one reported for the RTVue device, for which a CoV in the central zone of 1.07% has been reported in unoperated eyes [4]. The repeatability of the REVO NX ETMs found in this study cannot be directly compared to the values reported in the literature by other authors using REVO NX because they made maps based on a 5-mm averaged radial scan without an anterior adapter [17]. Like in other studies, the superior quadrants were found to be the thinnest while the inferior was the thickest [18].

The study has some limitations. First of all, the repeatability and reproducibility were assessed on the basis of a relatively small group of subjects, which did not include patients with corneal pathologies. Also, the parameters were not tested using a higher resolution averaging scanning protocol. Further study should aim to better estimate the limits of the simultaneous corneal topography and ETM applicability in corneal diseases. It would also be particularly valuable to evaluate the exact sensitivity and specificity of the REVO NX ETMs for detecting corneal diseases and their comparison with ETMs obtained from OCT data from other OCT devices (e.g., Avanti). Future studies will need to include larger populations, with different ocular conditions.

In conclusion, the study demonstrated high precision (intraobserver repeatability and interobserver reproducibility) of the epithelial thickness measurement using REVO NX. However, increasing the distance from the corneal center is accompanied by a slight decrease in precision. The study also demonstrated excellent spatial correlation between ETMs and corneal topography exams performed simultaneously, which stems from the fact that both map types are created from the same OCT data, they are based on the same 3D model, and they are ideally centered in the same way. This way of analyzing the surface, curvature, and thickness of the cornea and its epithelium may become a routine procedure in the near future.

## Figures and Tables

**Table 1:** Intraobserver repeatability results for ETMs using REVO NX based on three measurements taken by 2 operators in a sample of 40 eyes.

Sector	Operator	Mean ± SD	TRT	Sw	CoV (%)	ICC
Superior (*μ*m)	1st	51.53 ± 3.45	1.60	0.58	1.12	0.98
	2nd	51.27 ± 3.22	3.36	1.21	2.36	0.88
Superior-temporal (*μ*m)	1st	51.47 ± 3.28	1.89	0.68	1.33	0.96
	2nd	51.30 ± 3.00	3.89	1.40	2.73	0.82
Temporal (*μ*m)	1st	51.43 ± 3.26	1.82	0.66	1.28	0.97
	2nd	51.33 ± 2.99	3.36	1.21	2.36	0.86
Inferior-temporal (*μ*m)	1st	51.23 ± 3.09	2.77	1.00	1.95	0.91
	2nd	51.53 ± 3.12	2.09	0.75	1.46	0.95
Inferior (*μ*m)	1st	51.13 ± 3.04	3.12	1.13	2.20	0.89
	2nd	51.47 ± 3.07	2.37	0.86	1.66	0.94
Inferior-nasal (*μ*m)	1st	51.07 ± 2.98	3.51	1.26	2.48	0.85
	2nd	51.57 ± 2.99	2.53	0.91	1.77	0.92
Nasal (*μ*m)	1st	51.20 ± 3.06	2.53	0.91	1.78	0.93
	2nd	51.43 ± 3.05	2.77	1.00	1.94	0.91
Superior-nasal (*μ*m)	1st	51.17 ± 3.07	2.86	1.03	2.02	0.91
	2nd	51.73 ± 2.83	3.04	1.10	2.12	0.88
Central (*μ*m)	1st	51.50 ± 3.22	1.96	0.71	1.37	0.96
	2nd	51.43 ± 3.34	2.21	0.80	1.55	0.95
Superior_Out (*μ*m)	1st	51.13 ± 2.96	3.95	1.43	2.79	0.81
	2nd	51.67 ± 2.67	3.98	1.44	2.78	0.76
Superior-temporal_Out (*μ*m)	1st	51.20 ± 2.80	3.89	1.40	2.74	0.79
	2nd	51.80 ± 2.62	3.51	1.26	2.44	0.81
Temporal_Out (*μ*m)	1st	51.10 ± 2.87	4.08	1.47	2.88	0.78
	2nd	51.67 ± 2.44	4.11	1.48	2.87	0.69
Inferior-temporal_Out (*μ*m)	1st	51.53 ± 2.61	4.05	1.46	2.83	0.74
	2nd	51.87 ± 2.47	2.95	1.06	2.05	0.85
Inferior_Out (*μ*m)	1st	51.20 ± 2.80	3.58	1.29	2.52	0.82
	2nd	51.80 ± 2.62	3.51	1.26	2.44	0.81
Inferior-nasal_Out (*μ*m)	1st	51.93 ± 2.74	3.36	1.21	2.33	0.79
	2nd	52.03 ± 2.63	3.36	1.21	2.33	0.82
Nasal_Out (*μ*m)	1st	51.57 ± 2.58	2.26	0.82	1.58	0.92
	2nd	51.43 ± 3.00	2.99	1.08	2.10	0.89
Superior-nasal_Out (*μ*m)	1st	51.30 ± 2.20	4.05	1.46	2.85	0.64
	2nd	51.67 ± 3.24	3.61	1.30	2.52	0.87

**Table 2:** Interobserver reproducibility results for ETMs using REVO NX based on the first readings from each session taken by 2 operators in a sample of 40 eyes.

Sector	Mean ± SD	TRT	Sw	CoV (%)	ICC
Superior (*μ*m)	51.40 ± 3.31	2.63	0.95	1.85	0.92
Superior-temporal (*μ*m)	51.38 ± 3.11	3.07	1.11	2.16	0.87
Temporal (*μ*m)	51.38 ± 3.10	2.70	0.97	1.90	0.90
Inferior-temporal (*μ*m)	51.38 ± 3.08	2.48	0.90	1.74	0.91
Inferior (*μ*m)	51.30 ± 3.03	2.69	0.97	1.89	0.90
Inferior-nasal (*μ*m)	51.32 ± 2.97	2.95	1.06	2.07	0.87
Nasal (*μ*m)	51.32 ± 3.03	2.46	0.89	1.73	0.91
Superior-nasal (*μ*m)	51.45 ± 2.94	2.97	1.07	2.08	0.86
Central (*μ*m)	51.47 ± 3.25	2.00	0.72	1.40	0.95
Superior_Out (*μ*m)	51.40 ± 2.81	3.78	1.36	2.65	0.76
Superior-temporal_Out (*μ*m)	51.50 ± 2.70	3.59	1.30	2.52	0.77
Temporal_Out (*μ*m)	51.38 ± 2.66	4.06	1.46	2.85	0.69
Inferior-temporal_Out (*μ*m)	51.70 ± 2.53	3.36	1.21	2.34	0.77
Inferior_Out (*μ*m)	51.50 ± 2.70	3.59	1.30	2.52	0.77
Inferior-nasal_Out (*μ*m)	51.98 ± 2.66	3.36	1.21	2.33	0.79
Nasal_Out (*μ*m)	51.50 ± 2.78	3.66	1.32	2.57	0.77
Superior-nasal_Out (*μ*m)	51.48 ± 2.75	4.80	1.73	3.37	0.60

## Data Availability

The data are available on request.
